# Assessing the emissions of short sea international shipping: a case study of the Mytilini–Ayvalik route

**DOI:** 10.1007/s11356-023-30595-5

**Published:** 2023-10-26

**Authors:** Alexandros Kelmalis, Dimitrios F. Lekkas, Konstantinos Moustakas, Stergios Vakalis

**Affiliations:** 1 Department of Environment, Energy Management Laboratory, University of the Aegean, University Hill, 81100 Mytilini, Greece; 2 Department of Environment, Waste Management Laboratory, University of the Aegean, University Hill, 81100 Mytilini, Greece; 3 Unit of Environmental Science & Technology, School of Chemical Engineering, National Technical University of Athens, Zographou Campus, 15780 Athens, Greece

**Keywords:** Green shipping, Decarbonization, Emission analysis, CO_2_ accounting

## Abstract

Short sea shipping involves transporting passengers and cargo between European ports and coastal countries bordering Europe on enclosed seas. Challenges include aging fleets and environmental concerns, but efforts to reduce emissions are in progress through the use of low-sulfur fuels, hybrid and electric vessels, and improved energy efficiency. This study focuses on emissions from international short shipping routes between Mytilini and Ayvalik, using standardized emission factors to calculate CO_2_, SO_2_, NO_*x*_, PM, and HC emissions. The research aims to bridge knowledge gaps and provide insights into these emissions, identifying the main commercial ships on the route, analyzing their emissions, and discussing the findings. Overall, six ships completed the route, consuming an average of 60 L of fuel per hour per 100 HP, depending on factors like speed and total load. Notably, NO_*x*_ emissions are the highest, followed by sulfur oxides, with values exceeding 12.9 and 3.5 tons, respectively. As anticipated, cruising is the shipping practice with the highest energy footprint, amounting to 695 MWh. An essential discovery is that hoteling plays a significant role in energy consumption and emissions, accounting for 152 MWh out of the total 881 MWh consumed across all shipping practices. The potential adoption of hydrogen as a fuel holds the promise of substantial reductions in greenhouse gas emissions, enhancements in air quality, and noise pollution mitigation.

**Supplementary Information:**

The online version contains supplementary material available at 10.1007/s11356-023-30595-5.

## Introduction

Short sea shipping, according to the European Integration Archives, refers to the transport by sea of passengers and freight between ports located in Europe or between ports based in non-European coastal countries bordering Europe on enclosed seas but without crossing oceans (COM (99) 317 final, 1999). As typical short sea shipping routes are defined the shipping transports within areas like the Baltic Sea, the Black Sea, and short routes in the Mediterranean Sea. The term covers domestic and international shipping, including feeder service provision, to and from islands, rivers, and lakes across the coast. The limited distance, often less than 100 miles, between nearby EU countries gives true short sea voyages within the EU an “international voyage” classification that contributes to a separate, more complicated customs regime. For instance, a trip from Antwerp to Rotterdam is currently an international trip, despite being less than 100 miles long, whereas a journey of 2000 miles from Houston to New York is purely domestic. At the same time, the fractured coast gives complete access to non-EU flagged vessels to compete for intra-EU cargo in direct competition with flagged Indian, Chinese, Russian, or even US vessels, all of which enjoy exclusivity in their own domestic waters. In addition, short sea shipping also means “co-modality” as coastal sea travel either begins or finishes with transport by bus, inland barge, or train. For the transport of Project Cargo, short sea shipping and inland waterways are highly critical as the indivisible loads are shipped as there are no mounting or demounting of units. That implies a considerable saving on rigging and payroll costs (Miloslavskaya et al. 2019).

The industry of short sea shipping is known to be highly competitive. Two main tactics are used as per UNCTAD (2019). On the one hand, there are traditional standard port-to-port transport tactics, primarily for bulk transport. On the other hand, there is the traditional standardized port-to-port transport, i.e., multimodal transport for general cargo. It should be denoted that the -in some cases- moderate economic returns of short sea shipping can be compensated by the fact that multimodal approaches allow container fleets to be operated more effectively, encourage repositioning, and may provide the potential to eliminate trips with empty vessels (Miloslavskaya et al. 2019). It should be denoted that short sea shipping is adversely affected by the competition with other modes of transport, which are highly subsidized or can evade several direct and indirect costs. Therefore, promoting a fair playing field in both forms could support the short sea maritime industry. Short sea shipping can be an environmentally sustainable and inexpensive option as it does not to demand significant investment in infrastructure and enhances the intra-community transport needs of people. Nevertheless, there is growing evidence today that the European Short Sea fleets have an aging problem, an issue that has been neglected (Tzannatos 2005). Wijnolst and Waals (2005) showed that more than 38% of ships with sizes between 500 and 10,000 gross registered tonnage (GRT) were 25 years of age or older. The equivalent figure at the time was less than half for the deep-sea fleet. It is expected that the environmental policies under consideration in the various international national bodies will have a very strong effect on how ships will be designed and run in the not-too-distant future. Regardless of the option selected, ships will be exposed to a charging scheme which will penalize air emissions in a manner that will not be changed from what is currently applied to industrial polluters. As it has become clear so far, the shipping industry is facing increasingly stringent regulations on emissions and sulfur fuel restrictions, with which it has to adapt. These changes, which directly affect the heavy oil fuels area, have drawn the attention of shipowners to the possibilities of offering low-sulfur fuels. These costs would have a clear effect on each carrier’s operational expenditures, preferably in a fair way such that no distortions are implemented (IMO 2020).

Vessels used in short sea shipping can be described as a modern polluter. Because of numerous administrative systems, shipowners are compelled to put resources into advances that are still generally unrewarding according to a monetary perspective; however, according to a natural perspective, new cross breed and electric-controlled vessels in marine transportation are the principal need to lessen the harmful emissions. There are countless short sea shipping lines all over the planet which, as a result of the public interest, should give traveler transport no matter what their benefit. As per the Third International Maritime Organization (IMO) Greenhouse Gas Study, sea transport contributes around 940 million tons of CO2 each year, or around 2.5% of worldwide ozone depleting substance outflows (Olmer et al. 2017). Assuming no prompt move is made to diminish harmful emissions, the International Maritime Organization predicts that this figure will ascend to as much as 15% by 2050 (Van Themaat and Reuder 2018). The specific sum is not set in stone by the future social and monetary circumstances. The IMO Sulphur Regulation marks a significant change in the maritime industry due to the reduced allowable sulfur content in marine fuels from 3.5 to a much lower 0.5% on a global scale. At the same time, the NO_*x*_ emission limits set forth by the IMO are applicable to all engines and are determined based on an engine’s maximum operating speed. It is important to note that Tier I and Tier II standards are globally mandated, while Tier III standards are specific to currently designated *Emission Control Areas* (ECAs) for NO_*x*_.

Notwithstanding, outflows from delivery will increase in all scenarios. Ship contamination mindfulness is continually developing, and the impact of advanced media and informal organizations is prompting the quick utilization of administrative structures pointed toward lessening and controlling emanations from ships. The goal to build the energy productivity of short sea shipping to diminish emanations of SO_*x*_, NO_*x*_, CO_2_, and PM (MARPOL Annex VI) prompts the presentation of new innovations (Čampara et al 2018). For numerous years, the fundamental fuel utilized in delivery was a weighty fuel oil; however, with electric and half and half ships entering the market, the comprehension of vessels has changed.

This study focuses on the international short shipping routes from Mytilini (Greece) to Ayvalik (Turkey) due to the frequent international routes and the relative short distance, i.e., 36 nautical miles. International maritime shipping contributes to around 800 million tons of CO_2_ emissions annually, yet it operates outside the regulatory framework of the Paris Agreement. The United Nations’ International Maritime Organisation (IMO) is tasked with spearheading efforts to curtail CO_2_ emissions in this sector, but reaching a consensus on concrete regulations and limitations has proven to be challenging. At the same time, accounting CO_2_ emissions of international maritime shipping routes is another challenge and this study aims to set the framework for addressing the issue. A research gap that is being addressed is the emissions of action during the route, i.e., cruising, maneuvering, and hoteling, along with the contribution of each action to the total emissions. The international short sea shipping routes of Lesvos island are quite important for the local economy but also to the overall shipping practices of Greek maritime industry. The scope of the manuscript is to identify the main commercial ships that perform the route, account for the emissions of their shipping practices, and discuss the outcomes of the analysis.

## Materials and methods

### Emission factors and utilized databases

The primary focus of this research revolved around evaluating the environmental consequences associated with the main commercial shipping routes leading to the Mytilini port within a specific month. To ensure a comprehensive assessment of the port’s regular summer operations before the COVID-19 pandemic, the analysis concentrated on June 2019. Initially, these routes were documented with assistance from Marine Traffic S.A., a company that supplied detailed data concerning the number of ship arrivals, the deadweight tonnage (DWT), draught, distance traveled, and ship types. The study utilized publicly accessible information to ascertain the engines used by each shipping vessel, all of which employed heavy fuel oil. Additionally, independent research on average fuel consumption per nautical mile was conducted. Combining this information with the routes provided by Marine Traffic S.A. and publicly available data, the study then calculated the total fuel consumption for the relevant ships. Marine Traffic S.A. maintains a comprehensive and valuable database that provides detailed information on global ship movements and related maritime data. This database serves as a vital resource for various stakeholders, including researchers, maritime professionals, and port authorities (Barbopoulos et al. 2019). Researchers have leveraged the wealth of data from Marine Traffic S.A. to study vessel traffic patterns, analyze maritime transportation efficiency, and assess environmental impacts. Port authorities have utilized this database to enhance port management and optimize vessel traffic flow (Perivoliotis et al. 2019). Moreover, maritime professionals rely on Marine Traffic S.A.’s database for real-time vessel tracking, voyage planning, and situational awareness at sea. The reliability and accuracy of the database make it an indispensable tool in the maritime industry (Anbarci et al. 2021).

To calculate the overall emissions of CO_2_, SO_2_, NO_*x*_, PM, and HC for both national and international shipping routes under investigation, the study utilized standardized emission factors sourced from two reports: the technical report “Air Pollutant Emission Inventory Guidebook” published by the European Environment Agency (EMEP/EEA 2016) and the Entec report. The emissions were quantified based on the information provided in the following paragraph. Table 1 in this study presents the emission factors expressed in grams per kilowatt-hour (g/kWh) as published in the EMEP/EEA report.

**Table 1:** Emission factors of Mediterranean shipping vessels in g/ kWh (EMEP/EEA 2016)

Emissions	Main engine		Aux. engine
g/kWh	Cruise	Maneuvering	All activities
NO_*x*_	12	10.6	13.9
SO_2_	4.1	4.5	4.3
CO_2_	645	710	690
HC	0.5	1.5	0.4
PM	0.9	1.5	0.3

The main focus of this study was to examine the emissions produced by the major routes taken by passenger, ro-ro cargo, and ro-ro/passenger vessels that regularly arrive at the port of Mytilini according to a fixed schedule. These specific routes account for over 85% of the total number of arrivals and more than 90% of the total deadweight tonnage. To ensure the confidentiality of ship operators and their respective companies, specific values pertaining to the shipping vessels are not disclosed in the study.

### Methodology and calculations for emission analysis

Table 2, sourced from the EMEP/EEA report, provides information on the load percentage and operational times of both main and auxiliary engines. These values are crucial for converting the nominal power of engines on each ship into adjusted values that correspond to specific actions such as cruising, maneuvering, and hoteling. The analysis specifically focuses on heavy fuel oil as the fuel of consideration. While maintaining the anonymity of the shipping vessels, it can be stated that passenger ships have an average main engine power of 7668 kW, ro-ro cargo ships have an average main engine power of 11600 kW, and small (short sea) passenger ships have an average main engine power of 1300 kW. The power output and operational hours are adjusted accordingly for each action, referring to the values provided in Table 2.

**Table 2:** Percentage of engines operation (ME: main engine; AE: auxiliary engine)

	% load ME	% time ME operation	% load AE
Cruise	80	100	30
Maneuvering	20	100	50
Hoteling	20	5	40

**Fig. 1:**
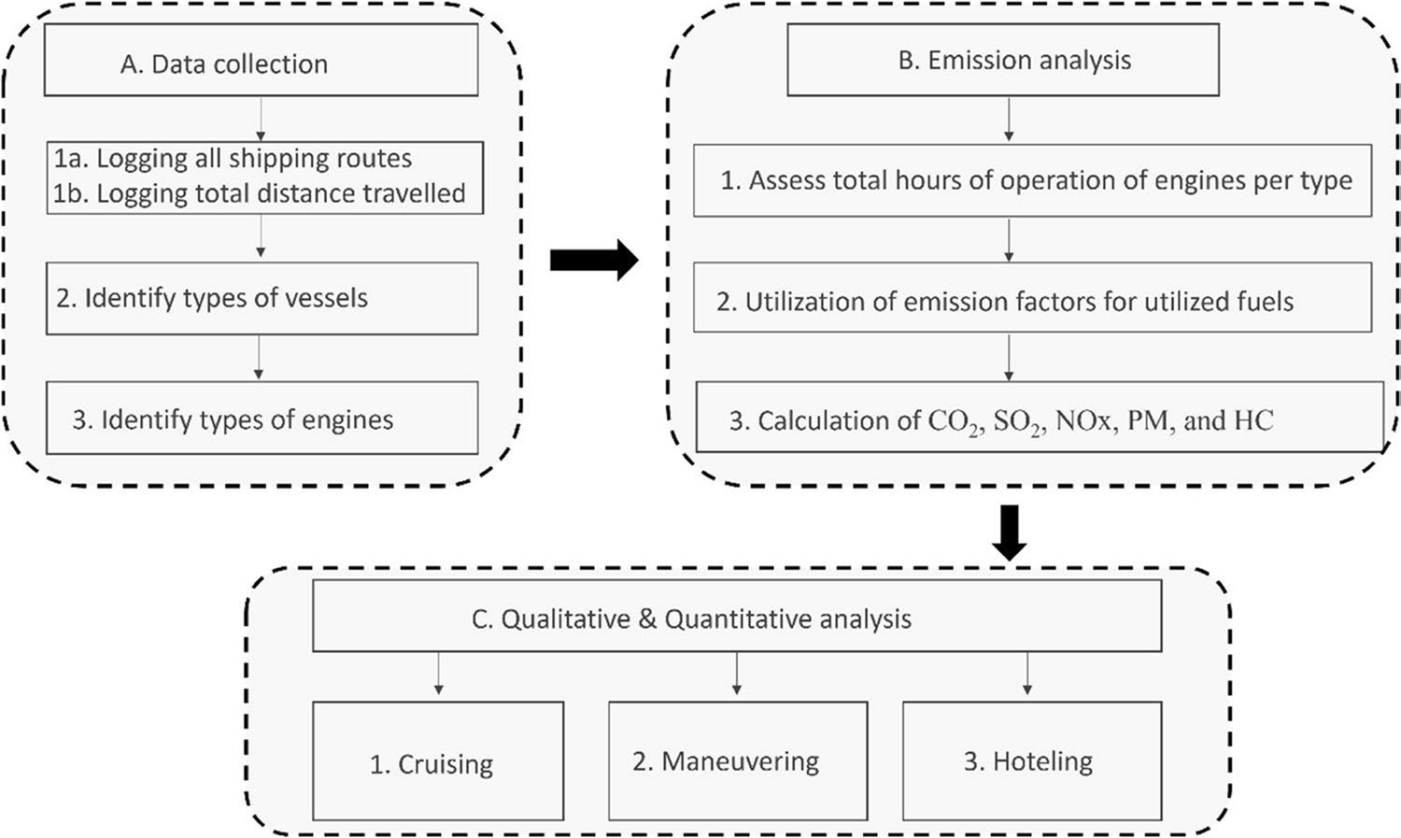
The developed methodology for assessing the emissions from short sea shipping

The flowchart of the methodology is being presented in Fig. 1. Overall, the methodology uses as baseline a meticulous logging of all shipping activities in respect of type of ship, time of arrival, time of departure, and other factors mentioned previously like the type of vessel and the DWT. An additional factor was the distance covered by each shipping route. Although, the start and endpoints were the same, i.e., Mytilini–Ayvalik, all the individual shipping routes were logged individually in order to account even for minor deviations in the routes. Onsite research was utilized to identify the types of engines and the types of fuels that were utilized. The study utilized emission factors for CO_2_, SO_2_, NO_*x*_, PM, and HC that were developed specifically for the types of shipping vessels that are used for the investigated route. The times of operation for each shipping activity, i.e., cruising, maneuvering, and hoteling, were logged during the initial stage of the research along with all the other parameters that were being logged.

## Results and discussion

**Fig. 2:**
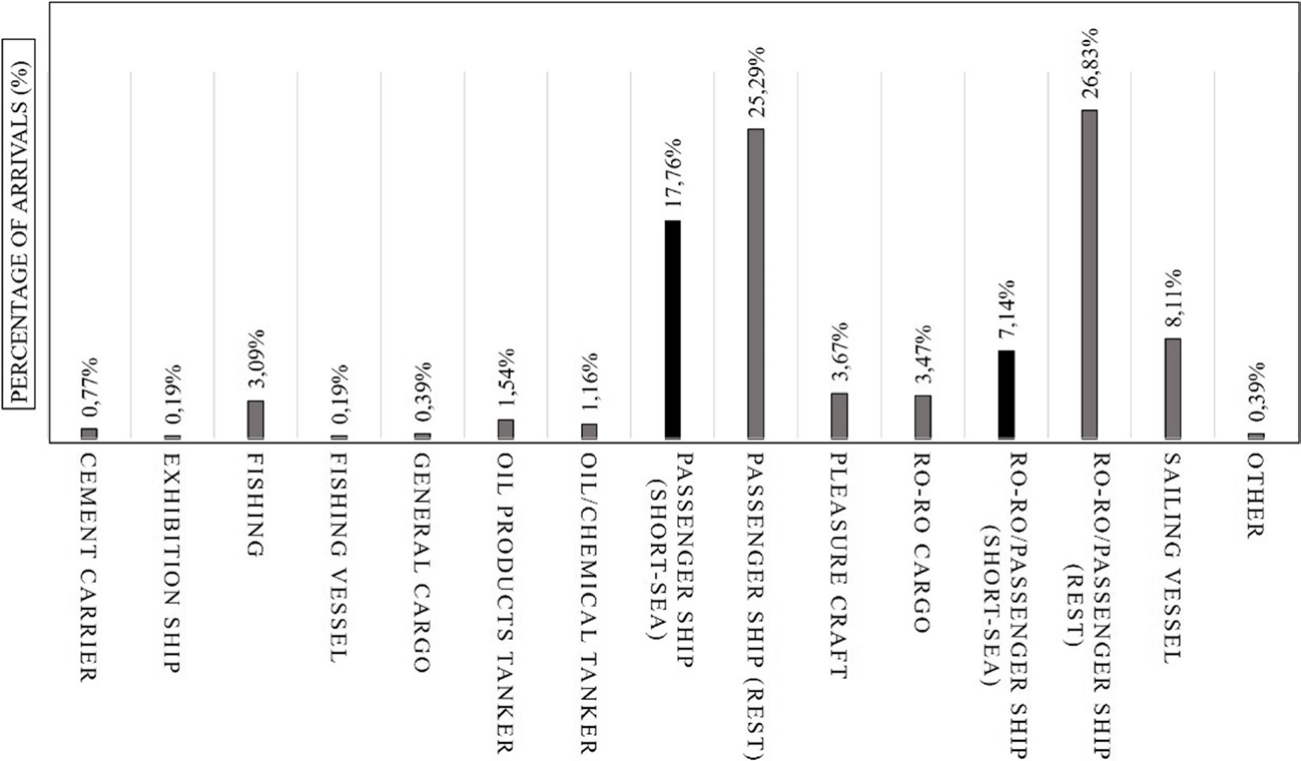
Total arrivals per shiiping vessel type at the port of Mytilini (July 2019)

The applied methodology aims to provide valuable insights on a quantitative and on a qualitative level. Thus, the results presented in the framework of this study initially aim to provide the full picture in respect of the total arrivals at the port of Mytilini, to focus deeper on the specific types of vessels that make the route Mytilini–Ayvalık and to specifically calculate the emissions from each individual shipping engine and each individual route. Figure 2 illustrates the distribution of arrivals by type at the port of Mytilini in June 2019. Passenger ships constituted the largest percentage, representing 42.4% of the total arrivals. Following closely behind were ro-ro/passenger vessels, accounting for 33.9% of the total arrivals. ro-ro cargo ships, on the other hand, comprised approximately 3.5% of the total arrivals. These three types of ships, with regular schedules, constituted the majority of the arrivals, amounting to approximately 80% of the total. It is worth noting that there are other ships involved in essential supply services such as the delivery of heavy fuel oil and cement to the island. However, Fig. 1 does not include military shipping vessels or other non-scheduled shipping activities, which may contribute to a different total arrival count.

Short sea shipping routes play a significant role in maritime transportation, and understanding the emissions associated with these routes is crucial for environmental assessments. Several studies have examined the emissions from short sea shipping routes, providing valuable insights into their environmental impact. Research conducted by Jalkanen et al. (2018) investigated the emissions of greenhouse gases (GHGs) from short sea shipping in the Baltic Sea region. Their findings revealed that CO_2_ emissions accounted for the majority of GHG emissions, highlighting the importance of addressing carbon emissions in this sector. Additionally, a study by Psaraftis and Kontovas (2019) examined the emissions from short sea shipping in the European Union, emphasizing the need for efficient operational practices to reduce emissions and improve environmental performance. Furthermore, research conducted by Benveniste et al. (2020) focused on the air pollution impacts of short sea shipping in the Mediterranean region, highlighting the significance of reducing emissions of air pollutants such as sulfur oxides (SO_*x*_) and nitrogen oxides (NO_*x*_). These studies collectively contribute to our understanding of emissions from short sea shipping routes and provide insights into strategies for mitigating their environmental impact. Table 3 shows the mechanical characteristics and the shipping vessels that connect Mytilini–Ayvalik. After detailed research, it became clear that all routes are been carried out by the Turkish Ferry Company Turyol. The general information about the main engine power, the auxiliary engine power along with the number of total routes for each ship, are provided below.

**Table 3:** Operational information—examined shipping vessels

	ME power (kW)	AE power (kW)	Total routes	Calendar days of operation/month
Ship A	1491	125	37 × 2	30
Ship B	3123	148	15 × 2	14
Ship C	3002	125	23 × 2	21
Ship D	1233	411	26 × 2	26
Ship E	822	160	27 × 2	25
Ship F	1268	634	1 × 2	1

The information provided includes details about several ships operating on the Ayvalik–Mytilini route. The first ship, Lesvos (IMO: 9323924), is a ro-ro/passenger ship built in 2004, sailing under the flag of Turkey. It has a carrying capacity of 332 gross tonnage, a summer DWT of 180 t, and current draught of 2.3 ms. Another ship, Nazli Jale (IMO: 8506945), is also a ro-ro/passenger ship, built in 1986, with a carrying capacity of 288 gross tonnage, a summer DWT of 50 t, and current draught of 1.3 m. Similarly, Esref Jale (IMO: 9053701) is a ro-ro/passenger ship built in 1992, with a carrying capacity of 269 gross tonnage, a summer DWT of 50 t, and current draught of 1.8 m. Seda Jale (IMO: 8987591) is a passenger ship built in 2004, with a carrying capacity of 367 gross tonnage, a summer DWT of 167 t, and current draught of 2.3 m. Lastly, Kaptan Ilyas Mert (IMO: 9697533) and Kaptan Sevket iyidere 1 (IMO: 9089176) are passenger ships with carrying capacities of 453 gross tonnage and 329 gross tonnage, respectively. These ships have varying fuel consumption rates depending on factors such as speed, with an average fuel consumption of 60 L/hour/100HP, which may increase by 20% at higher speeds. Hoteling times and maneuvering times are crucial for calculating in-port emissions. However, this study also aims to assess emissions during cruising. The specific values related to hoteling-idling times, maneuvering times, and cruising times are presented collectively in Table 4. Combined with the factors provided in Tables 1 and 2, these values are used to calculate both in-port and open-sea emissions.

**Table 4:** Routing times of examined shipping vessels

	Cruise (h)	Manoeuvring (h)	Hoteling (h)
Ship A	1.37	0.15	0.3
Ship B	0.75	0.15	0.3
Ship C	0.75	0.15	0.3
Ship D	1.43	0.15	0.3
Ship E	1.47	0.15	0.3
Ship F	1.50	0.15	0.3

**Fig. 3:**
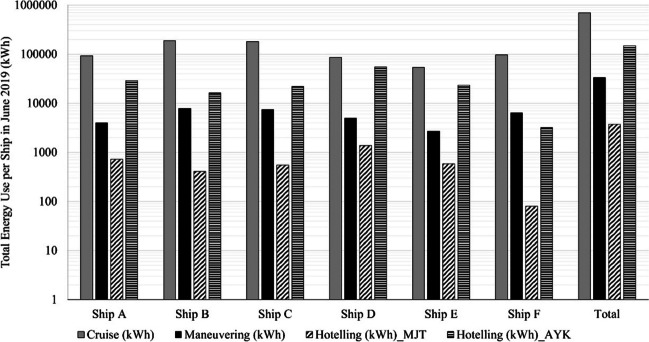
Total energy comnsumption and per examined shipping vessel type for the route Mytilini–Ayvalik (July 2019)

Figure 3 presents the energy consumption of each examined shipping vessel by type of action, i.e., cruising, hoteling, and maneuvering. The energy consumption of ships in short sea shipping routes has been a subject of extensive research, aiming to improve efficiency and reduce environmental impacts. Studies have explored various aspects of energy consumption in this context. For instance, a study by Hildebrandt et al. (2017) examined the energy consumption patterns and optimization potential of short sea vessels, emphasizing the importance of energy-saving measures. Saarela et al. (2018) analyzed the energy efficiency of short sea shipping in the Baltic Sea region, considering factors such as vessel size, speed, and cargo volume. Andersson et al. (2019) investigated energy consumption in short sea shipping, focusing on vessel design and operational factors.

**Fig. 4:**
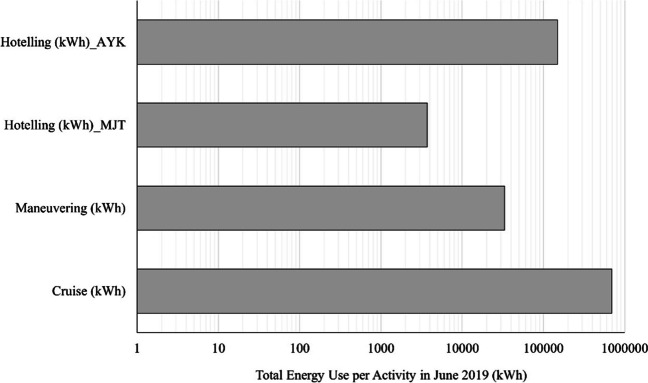
Total energy consumption for all examined shipping vessels for the route Mytilini–Ayvalik (July 2019)

Figure 4 presents the total energy consumption from all the shipping vessel combined for a whole calendar month. An interesting result—and one of the most significant results if this study—is that while cruising is consuming the most energy for all the examined ships, hoteling also has a significant impact and should be co-assessed as a significant contributor of emissions and of energy use. Goulielmos et al. (2020) assessed the energy efficiency of short sea container shipping, highlighting the role of ship design and operational practices. Wergeland et al. (2021) evaluated the energy consumption of short sea vessels in the Norwegian transport system, identifying opportunities for energy savings. These studies collectively contribute to a better understanding of energy consumption in short sea shipping and provide insights for achieving more sustainable maritime transportation practices. A point to be made is that the optimal design of short sea shipping routes in accordance to the specific shipping vessel can potentially reduce significantly the energy consumption and the emissions of the sector.

**Fig. 5:**
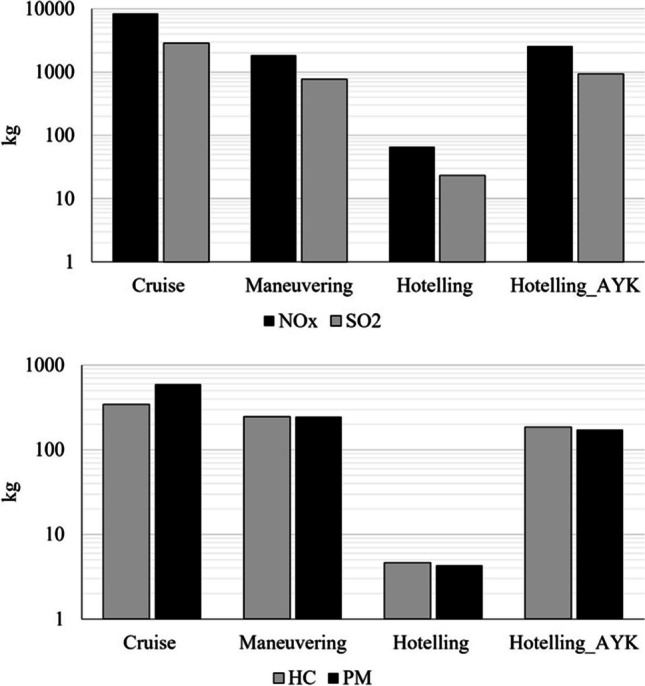
Total emissions for all examined shipping vessels for the route Mytilini–Ayvalik (July 2019)

Figure 5 presents the total emissions from the examined ships that did the route Mytilini–Ayvalık during July 2019. Nitrogen oxide (NO_*x*_) emissions are the emissions with the highest amount of production followed by sulfur oxides. Nitrogen oxides (NO_*x*_) emissions in short sea shipping have been a significant concern due to their environmental impact. Lutzhoft et al. (2019) examined the impact of ship speed and engine load on NO_*x*_ emissions in short sea shipping routes. The authors found that higher speeds and increased engine loads contribute to higher NO_*x*_ emissions. Another study by Baldauf et al. (2020) focused on the characterization of NO_*x*_ emissions from short sea shipping vessels, analyzing the effects of different operational and technical parameters. They emphasized the importance of adopting emission control measures and technologies to reduce NO_*x*_ emissions. Additionally, a study by Xu et al. (2021) evaluated the effectiveness of using exhaust gas recirculation (EGR) systems to mitigate NO_*x*_ emissions in short sea shipping. The authors found that EGR systems can significantly reduce NO_*x*_ emissions under various operating conditions. These studies contribute to understanding the factors influencing NO_*x*_ emissions in short sea shipping and provide insights for developing strategies to minimize their environmental impact.

Sulfur emissions in short sea shipping have been a significant environmental concern due to their impact on air quality and human health. Several studies have examined sulfur emissions in this sector and proposed strategies for mitigation. Apostolopoulou et al. (2020) investigated sulfur emissions from short sea shipping vessels in the Mediterranean Sea. The authors analyzed the effect of different fuel sulfur content and emission control technologies on sulfur emissions. They highlighted the importance of adopting low-sulfur fuels and implementing exhaust gas cleaning systems to reduce sulfur emissions. Stournaras et al. (2019) focused on assessing the impact of the sulfur emission control area (SECA) regulations on short sea shipping in the Baltic Sea. The authors found that SECA implementation significantly reduced sulfur emissions and improved air quality in the region. Additionally, a study by Psaraftis et al. (2018) evaluated the environmental and economic implications of different sulfur emission reduction strategies for short sea shipping. The authors assessed the feasibility and cost-effectiveness of using low-sulfur fuels, exhaust gas cleaning systems, and alternative energy sources.

**Fig. 6:**
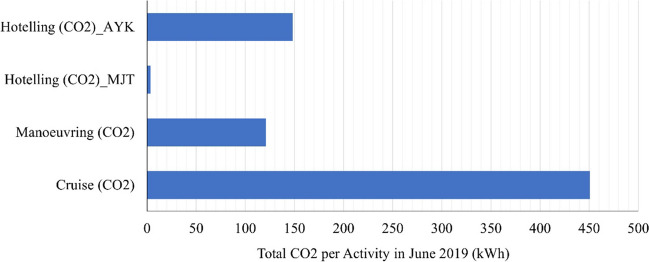
Carbon dioxide emissions for all examined shipping vessels for the route Mytilini–Ayvalik (July 2019)

Figure 6 presents the CO_2_ emissions from the examined route. CO_2_ emissions are in the center of conversation due to their contribution to climate change. A study by Cullinane et al. (2020) examined the factors influencing CO_2_ emissions in short sea shipping and identified operational and technological measures for CO_2_ reduction. The authors emphasized the importance of optimizing ship speed, route planning, and adopting energy-efficient technologies. Another study by Tsitsifli et al. (2019) assessed the CO_2_ emissions from short sea shipping routes in the Mediterranean Sea and proposed a methodology for calculating emissions based on ship characteristics and operational data. They highlighted the need for accurate emissions measurement and monitoring to support effective mitigation strategies. Additionally, a study by Wijnolst et al. (2018) investigated the potential of alternative fuels and propulsion systems to reduce CO_2_ emissions in short sea shipping. The authors assessed the environmental and economic feasibility of LNG, biofuels, and battery-powered vessels as viable alternatives. These studies contribute to understanding CO_2_ emissions in short sea shipping and provide valuable insights for developing sustainable and low-carbon shipping practices.

Atmospheric contamination is the greatest challenge for commercial shipping today as it threatens to undo the gains enjoyed by longer travel distances after China and India joined the WTO as a result of anti-pollution travel expenses. If environmental protection-based levies tend to be important, it would have the same effect as import levies that would hit items coming from longer distances. A large proportion of the short sea fleet currently operating intra-EU trade is over 25 years old, the oldest ships apparently carrying Russian, Norwegian, Greek, and Turkish flags. Ship age not only affects the cost of shipping mostly through the effect of the maintenance and repair bill but also through the effect of the maintenance and repair bill (Corres & Psaraftis, 2005). It is a costly endeavor to replace old ships with new, replacing whole pieces of a huge fleet, such as the short sea fleet is a mammoth process that will take at least 25–30 years to complete. Therefore, in order to be able to get environmentally friendly ships serving in EU trade, the EU’s attitude about shipbuilding and, of course, the scrapping of the shipbuilding directives will need to be modified, with the exception of funds. At this point, it is important to note that on a study conducted by Becker et al. (2008), comparing the costs of manufacturing and upgrading short and deep sea ships with regards to emissions, it was found that it is more expensive to care for based on the fact that most shipping companies give their full attention to their deep sea vessels and this is a wide phenomenon, nor restricted in the EU (Becker et al 2008). Most of the short sea flee as mentioned previously is quite old, and companies in the maritime industry do not wish to upgrade these vessels since there is no such support from EU (Corres and Psaraftis 2005).

The outcomes of this study can be used as a blueprint for assessing the environmental impact of other international short sea shipping routes/cases. In accordance with Mannarini et al. (2021) on the matter of emissions of short flee routes in Adriatic, they found that the Adriatic area is a significantly busy area for international short sea routes since it is on a close proximity with Italy, Montenegro, Croatia, and Albania. Even though the ships used to carry out these routes account for the 3% of the total, they do have significantly high emissions, nearly 10%. That shows us that there is a need for action. Alongside the International transportations by cargos etc., IMO should also include the International short sea routes as well since the emission of these ships is quite high (Mannarini et al. 2021). Additionally, Luttenberger et al. (2014) on a study conducted for the purpose of calibrating the emissions produced by Croatia’s short sea flee, they found that the substructure for minor emissions on these ships is quite low and undersized, even though Croatia is quite popular and significant when it comes to international short sea routes (Luttenberger et al. 2014). Lastly, Christodoulou and Woxenius (2019) underline the issue on absence of accountancy on the matter of sustainability of short sea international routes in the Mediterranean area by shipping companies and organizations of the maritime industry (Christodoulou and Woxenius 2019).

Future studies ought to consider how much non-renewable energy sources are consumed and the CO_2_ outflows created in the development of comparable power and the hydrogen expected to keep up with shores. The pattern of advances lies in power capacity innovation, the fruitful revelation of new materials for battery makes, and the acknowledgment that the focal point of worldwide industry improvement is on more effective types of sustainable power. The development of electric ships and hydrogen fuel, which creates power to charge batteries on electric ships, is conceivable through the utilization of existing advances (Rutherford et al 2020). Contrasted with marine diesel vessels, electric ships lead to a decrease of CO_2_ outflows by up to 90%, including altogether lower NO_*x*_, SO_*x*_, and PM discharges and working expenses by up to 80%. The utilization of hydrogen as a fuel can make a huge commitment to decreasing ozone depleting substance discharges, as well as further developing air quality and lessening commotion. Along these lines, with the backing of the European Commission, hydrogen is progressively tracking down its direction into all methods of transport and entering the European Union market.

## Conclusions

This study combined the individual data logging for all the international short shipping routes from Mytilini to Ayvalik and combined them with specialized emissions factors and engine parameters in order to calculate the overall CO_2_, SO_2_, NO_*x*_, PM, and HC emissions during the examined route. On a second level, qualitative analysis identifies the contribution of each shipping practice to the energy consumptions and the total production of emissions. Six ships completed the route, consuming an average of 60 L of fuel per hour for every 100 HP of engine power. On a qualitative basis, energy consumption for cruising was assessed to be at the level of 695 MWh. A notable point is that the combined energy consumption of hoteling for the two ports of Mytilini and Ayvalık exceeded the level of 152 MWh for the month of July, and thus, hoteling plays a significant role on the total accounting of energy consumption and emissions. Nitrogen oxides with 12 tons per month were identified to be the emissions with the highest production, followed by sulfur oxides that exceeded 3 tons per month for all the activities.

### Supplementary Information

Below is the link to the electronic supplementary material.

Supplementary file1 (DOCX 19 KB)

## Data Availability

The data utilized for this research are publicly available and can be provided to interested parties on request.
