# Supplementary material for: Assessing the emissions of short sea international shipping: a case study of the Mytilini–Ayvalik route
Source: Environ Sci Pollut Res Int. 2023 Oct 26;30(54):115496–505. doi: 10.1007/s11356-023-30595-5 (PMC10682216; doi:10.1007/s11356-023-30595-5)
Supplement: Supplementary file 1 — Supplementary file1 (DOCX 19 KB) [file 11356_2023_30595_MOESM1_ESM.docx]

Supplementary information

**Supplementary Table 1.** Analytical NOx and SOx emissions per ship for the for the route Mytilini – Ayvalik (July 2019).

|  | **Cruise** | **Maneuvering** | **Hoteling_MJT** | **Hotelling_AYK** |
| --- | --- | --- | --- | --- |
|  | **NOx (kg)** | | | |
| Ship A | 1112,32 | 243,64 | 13,58 | 543,24 |
| Ship B | 2252,74 | 501,33 | 8,66 | 325,22 |
| Ship C | 2161,75 | 480,65 | 12,13 | 446,36 |
| Ship D | 1057,40 | 225,07 | 21,22 | 848,65 |
| Ship E | 656,00 | 141,23 | 9,54 | 355,64 |
| Ship F | 1193,95 | 247,77 | 1,19 | 47,67 |
|  | **SO_2_ (kg)** | | | |
| Ship A | 378,34 | 102,32 | 4,87 | 195,00 |
| Ship B | 768,58 | 211,51 | 3,25 | 130,03 |
| Ship C | 737,66 | 202,94 | 4,59 | 183,76 |
| Ship D | 355,41 | 91,90 | 6,95 | 278,18 |
| Ship E | 221,80 | 58,54 | 3,22 | 128,87 |
| Ship F | 398,45 | 99,55 | 0,38 | 15,36 |

**Supplementary Table 2.** Analytical HC and PM emissions per ship for the for the route Mytilini – Ayvalik (July 2019).

|  | **Cruise** | **Maneuvering** | **Hoteling_MJT** | **Hotelling_AYK** |
| --- | --- | --- | --- | --- |
|  | **HC (kg)** | | | |
| Ship A | 45,67 | 33,39 | 1,05 | 42,01 |
| Ship B | 93,42 | 69,66 | 0,81 | 32,36 |
| Ship C | 89,70 | 66,93 | 1,17 | 46,96 |
| Ship D | 41,72 | 28,28 | 0,99 | 39,74 |
| Ship E | 26,40 | 18,60 | 0,54 | 21,59 |
| Ship F | 45,97 | 29,55 | 0,05 | 1,98 |
|  | **PM (kg)** | | | |
| Ship A | 80,60 | 33,32 | 0,99 | 39,78 |
| Ship B | 167,13 | 69,58 | 0,78 | 31,30 |
| Ship C | 160,58 | 66,86 | 1,14 | 45,58 |
| Ship D | 69,60 | 28,05 | 0,87 | 34,61 |
| Ship E | 45,34 | 18,51 | 0,49 | 19,52 |
| Ship F | 73,87 | 29,20 | 0,04 | 1,67 |
